# A Study on Characteristics Analysis and Countermeasures of Digital Sex Crimes in Korea

**DOI:** 10.3390/ijerph19010012

**Published:** 2021-12-21

**Authors:** Woochun Jun

**Affiliations:** Department of Computer Education, Seoul National University of Education, Seoul 06639, Korea; wocjun@snue.ac.kr; Tel.: +82-2-3475-2504

**Keywords:** digital sex crime, cybercrime, information and communication technology, information ethics, juvenile crime

## Abstract

In the modern knowledge–information age, various information and communication technologies provide us with many benefits and at the same time, bring various side effects such as cybercrime. The number of cybercrimes is increasing gradually, and in particular, the number of digital sex crimes has been increasing recently. In addition, digital sex crimes are becoming increasingly violent, so national measures are needed. In this study, statistical data at the national level were used to investigate the overall characteristics of digital sex crimes in Korea. First, statistical analysis shows that the victims are mainly women in their teens and 20s. Typical types of digital sex crimes are distribution of illegal contents and illegal filming, the perpetrators are mainly unknown, and digital sex crimes were less often recognized by others and more often by the victims themselves. Based on these results, countermeasures against various digital sex crimes are suggested.

## 1. Introduction

The dangers and seriousness of sex crimes cannot be overemphasized. This is because sex crimes that violate an individual’s right to sexual self-determination can cause physical and mental damage to the victim, and victims of sex crimes can suffer from trauma, for life in severe cases, due to mental stress and aftereffects. Moreover, recent sex crimes are becoming more serious, with the targets expanding beyond adults to minors, children, and infants, and unlike conventional sex crimes, these crimes are developing into a new non-physical type using various SNS and media online, without physical force such as assault and intimidation. This new type of digital sex crime is becoming more serious in that it does not end with illegal filming through production, distribution, consumption, and participation in illegal filming using digital devices, indeed, it is impossible to know where illegal films or videos spread [1,2,3].

The recent “Nth Room Case” using Telegram is a representative example of digital sex crimes becoming more intelligent and organized. According to [4], the Nth Room Case is a digital sex crime and sexual exploitation case that lured victims using messenger apps such as Telegram from the second half of 2018 to March 2020. The victims were a large number of minors, including middle school students, and a total of 1154 victims were confirmed at the end of the investigation in December 2020, of which 60.7% were in their 20s or younger. As of March 2020, the number of crime participants is at least 60,000 including illegal video holders and illegal distributors. As such, the production and distribution of digital sexual exploitation using new information and communication technologies such as the dark web are becoming more detailed, expanding, and the damage is serious, semi-permanent, and scalable, raising the need for specific measures to eradicate it. Specifically, it is urgent to come up with measures considering the seriousness of the issue, including various types of sexual crimes, such as producing, sharing, forcing, intimidating, indecent assault, rape, child abuse, and transmission and distribution of sexual exploitation videos.

As a result, comprehensive legislative, judicial, and administrative measures for evolving digital sex crimes and effective punishment are required, and the government has established measures to investigate digital sexual exploitation crimes, protect victims, and prohibit the distribution of sexual exploitation videos. The National Assembly of the Republic of Korea also enacted and passed various bills in 2020 to cope with new aspects of digital sex crimes that are becoming more intelligent and organized. Nevertheless, there are problems that have not been solved, such as the lack of stipulated laws on the concept of digital sex crimes, and various laws regulating contents related to digital sex crimes. However, there is a lack of unity in the details and punishments [5,6,7].

The purpose of this study is to investigate the current status of digital sex crimes in Korea and to suggest countermeasures against those crimes. Specifically, it is to identify the characteristics of digital sex crimes in Korea and to present specific countermeasures against digital sex crimes based on this characteristic analysis. In order to achieve this goal, first, an in-depth analysis was conducted based on the statistical data of digital sex crimes investigated at the national level to derive meaningful statistical facts. Based on these results, various measures to cope with digital sex crimes are proposed.

The rest of this paper is organized as follows. In Section 2, we introduce the definition, characteristics, and types of the digital sex crimes and introduce previous related works. In Section 3, we introduce the current status of digital sex crimes and present statistical analysis work of the digital sex crimes in Korea. In Section 4, implications of the results are discussed and various ways to prevent and reduce digital sex crimes in Korea are provided. In Section 5, we discuss conclusions of our work and present further research works in Section 6.

## 2. Related Works

### 2.1. Definition of the Digital Sex Crime

The term ‘online sexual violence’ began to formalize around the 2000s. In the early days, ‘online sexual violence’ was defined as ‘cyber sexual violence’ and was mixed with the concept of ‘cyber sexual violence’ that is defined as “an act that harms the other person, such as unpleasantness or pressure, even if sexual approach or suggestion is not explicitly presented”. During this period, online sexual violence was limited to only verbal expressions [8]. However, as digital technologies have developed rapidly, awareness of online crimes has also been raised. In other words, as pornography has been produced, distributed, and consumed online, beyond the offline boundaries, the perception that these types of crimes should be newly defined has become strong.

In Korea, the term ‘digital sex crime’ emerged after the so-called “Soranet” incident in 2015 [9,10]. A digital sex crime is informally defined as gender-based violence committed online and offline through digital devices and information and communication technology, and is also defined as a concept that seriously infringes on the sexual self-determination and moral rights of others in cyberspace by filming, storing, threatening, and distributing [5,8].

### 2.2. The Characteristics of the Digital Sex Crime

The characteristics of digital sex crimes are as follows [8]. The first characteristic is the persistence of damage. Digital crimes tend to neglect damage because of the perception that there is less direct damage than traditional crimes. However, once digital sex crimes are leaking online, they spread rapidly to an unspecified number of people, so the trauma of victims is more serious than that of ordinary sex crime victims. The main reason is that once the illegal content is distributed, it is almost impossible to delete it completely, so the victim feels anxious and frightened enough to undermine human dignity and personality and threaten survival. The second characteristic is the reproduction of gender violence and distorted gender awareness. Digital sex crimes can further strengthen the perception of “misogyny,” which has emerged since the 2010s in Korea. This is because the majority of victims of insulting remarks, sexual comments, and indecent jokes carried out online are women, and actual criminal acts tend to specifically target women, and threats and disparagement against women based on gender and sexuality are carried out online. The third characteristic of digital sex crimes is that there is a high possibility of social criticism or prejudice despite being victims. In particular, if the filmed footage is illegally distributed with the consent of the victim, it is easy to form a false perception of criticizing the victim. In other words, because it is a voluntary or consent-based filming, it is difficult to say that it has been unilaterally beaten by the perpetrator, and the view that the victim is responsible somehow is based on our society. The possibility of criticizing the victim in this way can delay the victim in reporting or taking action and cause the victim damage from which it is difficult to recover.

### 2.3. Types of Digital Sex Crimes

There are six types of digital sex crimes as follows [11]. The first type is ‘illegal photography,’ which involves filming parts of the body and sexual intercourse against the subject’s will or installing cameras in public places such as subways and toilets. The second type is ‘distribution and re-distribution’, which is the act of posting sexual footage on adult sites and social media, as well as sharing it with acquaintances, and distributing it against the will of the parties, regardless of whether they agreed to the photos being taken. The third type is ‘distribution threat’, which is an act of threatening to distribute sexual footage to others, as well as forcing sexual activity and additional filming under the pretext of intimidation. The fourth type is ‘synthetic production and distribution’, which is the act of sexually synthesizing and editing faces, body images, or voices, including so-called “deep fakes” [12]. The fifth type is ‘possession, purchase, and storage’, which is the act of possessing, purchasing, storing, or watching illegal films, and distributing materials including the sexual exploitation of children and adolescents. The last type is ‘sexual harassment in cyberspace’, which refers to the public distribution of personal information of victims of digital sex crimes or sexual defamation and insult.

### 2.4. Previous Works

The previous works on the current status of digital sex crimes in Korea are as follows.

Lee and Kang presented the current status of digital sex crimes in Korea and countermeasures, from the perspective of police practice [13]. That is, as a result of meeting digital sex crime victims in the police field and experiencing their damage cases, the types of digital sex crimes were identified as follows: sextortion (an act of suggesting obscene video chatting through a smartphone chatting application, recording the other party’s obscene acts or masturbation. Afterwards, a malicious code is planted on the victim’s smartphone to steal the contact information of the victim’s acquaintances, and the perpetrator then threatens to distribute the recorded video or photos to the victim’s acquaintances if money is not delivered), the crime of obscenity using communication media, illegally filming crimes using cameras, distribution of illegal films, and child and adolescent sexual exploitation. In addition, as countermeasures against digital sex crimes, they proposed the following: expanding the scope of non-disclosure investigations, expanding the scope of punishment for illegal filming and developing proof technologies, unifying departments on illegal filming crimes, and integrating victim protection and support systems.

From a criminal point of view, Kim examined the current status of digital sex crimes and proposed countermeasures in [14]. In particular, among the types of digital sex crimes, Kim focused on illegal filming crimes. According to the Supreme Prosecutors’ Office statistics and case analysis of camera-based crimes, 97.0% of victims of illegal filming crimes were women, and unless the crimes were discovered, the number of victims increased indefinitely. In addition, it was confirmed that all citizens, including women, could become victims due to the high frequency of damage in public places. Nevertheless, it has been identified that the level of punishment for related crimes has been very low so far. In addition, considering that the proportion of illegal filming of crimes is high among people in their teens and 20s, the need to improve media literacy education and awareness of digital sex crimes was emphasized for this age group. The effectiveness of strengthening the recently revised punishment regulations was emphasized, and it was also argued that the attention of related local governments is needed to prevent illegal filming crimes in public places in advance.

Kim and Park classified the types of digital sex crimes based on related laws, examined the status of digital sex crimes, and came up with policy improvement tasks to effectively cope with digital sex crimes, especially for illegal filming crime [5]. The improvement tasks were, first, systematic maintenance of laws and regulations related to digital sex crimes, second, solid protection of children and adolescents from digital sex crimes, and third, materialization of victim support for illegal filming, etc.

Um and Yoo investigated the status of digital sex crimes in children and adolescents in Korea [15]. According to their work, digital sex crimes against children and adolescents have different characteristics from other digital sex crimes because the perpetrators approach children and adolescents in a way that satisfies the needs of children and adolescents, forms intimacy, and causes digital sex crimes. On the surface they seem normal, but all acts by the perpetrator to approach or become close to children and adolescents are called grooming. Grooming using digital media such as online chatting, mobile messengers, and SNS are called digital grooming or online grooming. In particular, the authors argued that digital grooming is the most common type of crime against children and adolescents who spend a lot of time online.

Kim and Chung discussed the current status of illegal filming crimes and the limitations of existing relevant laws [16]. In addition, they argued that sexual violence crimes are propensity crimes and have a high probability of recurrence due to their highly habitual nature. They also argued that the criminal group was not homogeneous and did not show a single common characteristic. On the other hand, in responding to sexual violence crimes, resocialization through clear punishment and treatment was emphasized rather than strict punishment.

Kim investigated the status of cybersex crimes in Korea [17]. According to the work, overall sexual violence crimes were on the rise for the 10 years from 2006 to 2015. It is noteworthy that among sexual violence crimes, there is an increase in illegal filming. Illegal filming accounted for only 3.6% of all sexual violence crimes in 2006, but this proportion surged to 24.9% by 2015. Kim argued that this phenomenon was an increase in crime due to the increased daily use of smartphones and other electronic devices, increased awareness and sensitivity to sexual violence, active reporting of sexual violence crime, and trust in the crime damage protection system.

To summarize, previous studies have not used recent national-level integrated data and have not systematically described the characteristics of digital sex crimes. In contrast, this study attempts to more systematically and comprehensively describe the characteristics of digital sex crimes using the latest national-level digital sex crimes data.

## 3. The Current Status and Statistical Analysis of Digital Sex Crimes in Korea

In this section, we present the current status and statistical analysis of digital sex crimes in Korea.

### 3.1. The Current Status of the Digital Sex Crimes in Korea

In this section, we introduce the current status of digital sex crimes in Korea. The current status of digital sex crimes is based on statistical data published by the Women’s Human Rights Institute of Korea(http://www.stop.or.kr, accessed on 23 September 2021). The institute opened in 2009, and its purpose is to eradicate various forms of violence against women existing in our society, and, at the national level, to realize gender equality in society and contribute to the improvement of women’s human rights by preventing violence against women while supporting victims. The institute opened a Digital Sex Crime Victim Support Center in 2018 to establish a comprehensive support system for victims of digital sex crimes, including counseling support, deletion support, investigation and legal support linkage, and medical support linkage.

The institute has published statistics on the status of digital sex crimes in Korea since 2018. This paper introduces some of the digital sex crime statistics published by the institute [18]. First, Table 1 shows the age group of victims over the past three years from 2018 to 2020.

Table 2 shows the types of digital sex crimes over the past three years from 2018 to 2020. In the table, ‘distribution’ refers to cases where the illegal filming is distributed, and ‘illegal filming’ refers to cases where it was taken without consent. In addition, ‘distribution and threat’ is a case where threats have been made regardless of whether they are actually distributed, and ‘distribution and anxiety’ refers to cases of complaining of anxiety over the distribution. ‘Photo synthesis’ is a case in which photos are synthesized with sexual photographs without consent, and ‘cyberbullying’ refers to cases in which defamation or insults including sexual content are performed in cyberspace. Finally, ‘other’ means other types of violence such as stalking, sexual violence, and dating violence.

Table 3 shows the relationship of the perpetrator to the victim by year. In the table, ‘close relationship’ means a spouse, ex-spouse, lover, or ex-lover; ‘social relationship’ means a person who has an established relationship in work or social activities such as a school, workplace, institution, etc.; ‘family relationship’ means relatives such as parents, brothers, or sisters, excluding spouses; ‘temporary relationship’ means a chat partner or a person who has met only once; ‘stranger’ means a person whose perpetrator has been identified but has no knowledge of the victim; and ‘unknown” means a case where the perpetrator cannot be identified.

Table 4 shows the cognitive path of damage to digital sex crimes by year.

### 3.2. Statistical Analysis of Digital Sex Crimes

The purpose of this study is to analyze the characteristics of digital sex crimes in Korea and to suggest countermeasures against digital sex crimes based on the analyzed results. We performed statistical analysis on the current status of digital sex crimes. Based on previous statistical results, we present the following five hypotheses.

① There is no difference in damage from digital sex crimes according to gender.
② There is no difference in damage from digital sex crimes depending on age.
③ There is no difference in the frequency of occurrence according to the type of damage.
④ There is no difference in the frequency of occurrence according to the type of perpetrator of digital sex crimes.
⑤ There is no difference in the cognitive path of damage to digital sex crimes.

The digital sex crime statistics introduced in the previous section were analyzed using the SPSS (Statistical Package for the Social Science) WIN 25.0 program. *χ*^2^ (chi-squared) verification was performed as an analysis technique. The chi-squared test was used to analyze the difference between the groups that responded. The commonly used t-test is calculated as the mean and standard deviation, but the collected data are frequencies, so the differences were analyzed by chi-squared test.

(1) Victims of digital sex crimes by gender

As shown in Table 5, female victims (6985, 83.4%) accounted for the majority of the 8375 victims of digital sex crimes, and 1390 (16.6%) were male victims.

As can be seen in Table 5, there were far more female victims than male victims, by year, 1106 (84.1%) in 2018, 1832 (87.8%) in 2019, and 4047 (81.4%) in 2020. These differences are statistically significant (*χ*^2^ = 44.09, *p* < 0.001). Therefore, it can be seen that female victims accounted for the majority of victims in 2018, 2019, and 2020. Therefore, Hypothesis 1 is rejected. It is concluded that the majority of victims are women.

(2) Victims of digital sex crimes by age

As shown in Table 6, 3962 people (47.3%) were of unknown age, 1807 people (21.6%) in their 20s, 1636 people (19.5%) in their 10s, 608 people in their 30s (7.3%), 218 (2.6%) in their 40s and 144 (1.7%) in their 50s.

In Table 6, by year, excluding those of unknown age, 251 (19.1%) and 504 (24.1%) were in their 20s in 2018 and 2019, respectively, while 1204 (24.2%) were in their teens in 2020, showing statistically significant differences (*χ*^2^ = 233.02, *p* < 0.001). Therefore, victims in their teens and 20s accounted for the majority in 2018, 2019, and 2020. Therefore, it can be seen that Hypothesis 2 is rejected. It is concluded that, excluding those of unknown age, the majority of victims were in their 20 s or younger.

(3) Digital sex crimes by the type of crime

As shown in Table 7, 3938 cases (29.4%) of the 13,386 cases of digital sex crime victims were illegally filmed, followed by 3557 distribution cases (26.6%), 1823 distribution and anxiety cases (13.6%), 1529 distribution and threat cases (11.4%), 1290 other cases (9.6%), 687 cyberbullying cases (5.1%), and 562 photo synthesis cases (4.2%).

In Table 7, by year, distribution was the highest at 758 (33.1%) and 1213 (29.5%) in 2018 and 2019, respectively, while illegal filming was the highest at 2239 (32.1%) in 2020, showing statistically significant differences (*χ*^2^ = 403.99, *p* < 0.001). Therefore, it can be seen that distribution and illegal filming account for more than half of the digital sex crimes and that Hypothesis 3 is rejected. We can conclude that among the types of crime, ‘distribution’ and ‘illegal filming’ are the majority.

(4) Relationship between perpetrators and victims

As shown in Table 8, 3932 of the 8375 victims of digital sex crimes were of unknown relationship to the perpetrator (46.9%), followed by 1768 with a temporary relationship (21.1%), 1238 with a close relationship (14.8%), 822 were strangers (9.8%), 590 with a social relationship (7.0%), and 25 with a family relationship (0.3%).

In Table 8, by year, the number of unknowns was high with 498 (37.9%) and 651 (31.2%) in 2018 and 2019, respectively, while unknowns accounted for more than half with 2783 (56.0%) in 2020, showing statistically significant differences (*χ*^2^ = 964.46, *p* < 0.001). Therefore, it can be seen that the perpetrators of digital sex crimes account for a significant number of unknowns and that Hypothesis 4 is rejected. It is concluded that the majority of perpetrators are ‘unknown’.

(5) Digital Sex Crimes by the Cognitive Path of Damage

As a result of examining the current status of digital sex crime victims according to the cognitive path of damage, as shown in Table 9, of the total 8375 victims, 3480 (41.6%) were directly recognized by digital sex crime victims, followed by 3064 (36.6%) unknown and 1831 (21.9%) recognition by others.

As can be seen Table 9, in 2018 and 2019, direct recognition was the highest at 561 (42.7%) and 939 (45.0%), respectively, while in 2020, the unknown was the highest at 1901 (38.2%). Meanwhile, recognition by others was the smallest at 305 (23.2%) in 2018, 434 (20.8%) in 2019, and 1092 (22.0%) in 2020, showing statistically significant differences (*χ*^2^ = 21.14 and *p* < 0.001). This means that digital sex crimes were less often recognized by others and more often by the victims themselves. Therefore, Hypothesis 5 can be rejected. It is concluded that ‘recognition by others’ is less likely to occur in the cognitive path of damage.

## 4. Discussion

### 4.1. Analysis of Digital Sex Crimes

In this section, we analyze digital sex crimes based on the results of statistical analysis as follows.

As introduced in Section 3.1, digital sex crimes in Korea have been on the rise recently. This is presumed to be because the spread of digital devices is increasing with the development of information and communication technologies and smart technologies, and the use of digital devices is also increasing. According to the statistical analysis of digital sex crimes in Section 3.2, along with the rapid increase in digital sex crimes, the majority of victims are women, with most victims being in their 20s or younger.

In addition, distribution and illegal filming account for more than half of the various types of digital sex crimes because anyone can easily take pictures with a smartphone, and it is also easy to post on social media or adult sites. In addition, according to the statistics from [18], more than 85% of illegal filming contents are in the form of photographs making them convenient to take and store. The relatively high proportion of illegal photography means that digital sex crimes occur spontaneously rather than being planned, as in the case of cyberbullying [19].

On the other hand, the most statistically significant relationship between the perpetrators and victims of digital sex crimes was the unknown category. This is because it is possible to transmit and exchange information anonymously through online communication, and it is difficult to track down the perpetrator. In addition, lack of recognition of digital sex crimes by others means that the victim may suffer additional mental pain as well as the time and cost required to delete it.

### 4.2. Countermeasures against Digital Sex Crimes

In this section, we propose various countermeasures to reduce digital sex crimes in Korea as follows.

Digital sex crimes began to occur around the 2000s, but the crimes were not a big social problem, and legal punishment was also insignificant. It was the “Nth Room Case” that occurred in 2018 that alerted Korea to the seriousness of digital sex crimes and informed the need for a strong response. In 2018, the Women’s Human Rights Institute of Korea opened the Digital Sex Crime Victim Support Center under the Ministry of Gender Equality and Family and began comprehensive support such as deletion processing along with full-scale counseling for victims.

After the ‘Nth Room Case’, various digital sex crime-related punishment laws were enacted and began to take effect. However, strong law enforcement alone has limitations in responding to increasing digital sex crimes. In other words, legislation to respond to crimes caused by the development of new technologies takes time. Therefore, the fundamental measure is to prevent digital sex crimes through education rather than strong punishment.

Currently, in Korea, public education for digital sex crimes is not provided for elementary, middle, and high schools. There is no educational content in the curriculum on prevention, countermeasures, and counseling for digital sex crimes. Since it can take a considerable amount of time to include contents related to digital sex crimes in the public education curriculum, the most realistic way is to take charge of prevention, countermeasures, counseling, and education of digital sex crimes at national institutions. Currently, promotional materials and educational contents related to digital sex crimes are being developed, but national support is needed for systematic support in the future. Currently, the Digital Sex Crime Victim Support Center takes charge of the support, but it should be developed into a bigger institution for more organized support.

The countermeasures against the digital sex crimes proposed in this study are very general and realistic. Fundamental countermeasures against digital sex crimes should be implemented at the national level, and prevention is more important than follow-up, so we emphasized and suggested educational countermeasures.

## 5. Conclusions

Currently, we live in a knowledge and information society. Advances in information and communication technologies provide us with various benefits. Meanwhile, the development of information and communication technology is causing various side effects along with benefits. These representative side effects are evolving into various forms of cybercrime such as Internet addiction, personal information leakage, and copyright violations. Among these side effects, the issue of digital sex crimes has recently emerged. Anyone can easily and spontaneously become a perpetrator of digital sex crimes with simple digital devices such as smartphones, and anyone can become a victim.

In this study, we introduce the current status and suggest countermeasures of digital sex crimes in Korea. For objective analysis, statistical data at the national level were used to investigate and analyze the current status of digital sex crimes in Korea. As a result of statistical analysis, the following statistically significant conclusions were obtained. First, the majority of victims of digital sex crimes are women. Second, the majority of victims are teenagers or in their 20s. Third, most damage is through distribution and illegal filming. Fourth, the majority of perpetrators of digital sex crimes are unknown. Finally, digital sex crimes are less often recognized by others and more often by the victims themselves.

Strict punishment cannot be a fundamental countermeasure against digital sex crimes, and the best countermeasure is to prevent digital sex crimes through education. For this purpose, public education must provide full education to prevent digital sex crimes for elementary, middle, and high school students as early as possible. However, since education through curriculum revision takes time, provision of systematic support from institutions is needed at the national level.

## 6. Further Research Works

The future research works of this study are as follows. First, we need analyze more diverse data to identify the current status of digital sex crimes. Since the statistical data used in this study were collected only by victim reports, various cases of non-reporting were not reflected. Therefore, it is important to assess the current status using statistical data reflecting damage cases in a more active way. Second, analysis of the cause of digital sex crimes is needed. Knowing the cause will make it easier to come up with countermeasures against digital sex crimes. Third, various promotional materials should be developed to prevent digital sex crimes, as this can be done with less cost and effort than educational materials in public education.

## Figures and Tables

**Table 1:** Age Group of Victims by Year [18].

Year	Sex	10s	20s	30s	40s	Over 50s	Unknown
2018	Female	95	218	98	18	20	657
	Male	16	33	11	16	5	128
2019	Female	288	463	148	33	22	878
	Male	33	41	19	17	10	135
2020	Female	1007	863	267	77	36	1797
	Male	197	189	65	57	51	367

Unit: number of occurrences.

**Table 2:** Types of Digital Sex Crimes by Year [18].

Year	Distribution	Illegal Filming	Distribution and Threat	Distribution and Anxiety	Photo Synthesis	Cyber-Bullying	Other
2018	758	656	208	216	69	108	274
2019	1213	1043	354	557	144	273	530
2020	1586	2239	967	1050	349	306	486

Units: number of occurrences, duplicated responses allowed.

**Table 3:** Relationship of the Perpetrator to the Victim by Year [18].

Year	Close Relationship	Social Relationship	Family Relationship	Temporary Relationship	Stranger	Unknown
2018	309	136	5	198	169	498
2019	500	227	5	331	373	651
2020	429	227	15	1239	280	2783

Unit: number of occurrences.

**Table 4:** Cognitive Path of Damage by Year [18].

Year	Direct Recognition	Recognition by Others	Unknown
2018	561	305	449
2019	939	434	714
2020	1980	1092	1901

Unit: number of occurrences.

**Table 5:** Damage to Digital Sex Crimes by Gender.

Year	Woman	Man	Total	*χ* ^2^	*p*
2018	1106 (84.1%)	209 (15.9%)	1315 (100.0%)	44.09 ***	0.000
2019	1832 (87.8%)	255 (12.2%)	2087 (100.0%)		
2020	4047 (81.4%)	926 (18.6%)	4973 (100.0%)		
Total	6985 (83.4%)	1390 (16.6%)	8375 (100.0%)		

*** *p <* 0.001.

**Table 6:** Victims. of Digital Sex Crimes by Age.

Year	10s	20s	30s	40s	50s	Unknown	Total	*χ* ^2^	*p*
2018	111 (8.4%)	251 (19.1%)	109 (8.3%)	34 (2.6%)	25 (1.9%)	785 (59.7%)	1315 (100.0%)	233.02 ***	0.000
2019	321 (15.4%)	504 (24.1%)	167 (8.0%)	50 (2.4%)	32 (1.5%)	1013 (48.5%)	2087 (100.0%)		
2020	1204 (24.2%)	1052 (21.2%)	332 (6.7%)	134 (2.7%)	87 (1.7%)	2164 (43.5%)	4973 (100.0%)		
Total	1636 (19.5%)	1807 (21.6%)	608 (7.3%)	218 (2.6%)	144 (1.7%)	3962 (47.3%)	8375 (100.0%)		

*** *p* < 0.001.

**Table 7:** Types of Digital Sex Crimes by Year.

Year	①	②	③	④	⑤	⑥	⑦	Total	*χ* ^2^	*p*
2018	758 (33.1%)	656 (28.7%)	208 (9.1%)	216 (9.4%)	69 (3.0%)	108 (4.7%)	274 (12.0%)	2289 (100.0%)	403.99 ***	0.000
2019	1213 (29.5%)	1043 (25.4%)	354 (8.6%)	557 (13.5%)	144 (3.5%)	273 (6.6%)	530 (12.9%)	4114 (100.0%)		
2020	1586 (22.7%)	2239 (32.1%)	967 (13.8%)	1050 (15.0%)	349 (5.0%)	306 (4.4%)	486 (7.0%)	6983 (100.0%)		
	3557 (26.6%)	3938 (29.4%)	1529 (11.4%)	1823 (13.6%)	562 (4.2%)	687 (5.1%)	1290 (9.6%)	13,386 (100.0%)		

*** *p* < 0.001 (Unit: number of occurrences, duplicate responses allowed). where ①: distribution, ②: illegal filming, ③: distribution and threat ④: distribution and anxiety, ⑤: photo synthesis, ⑥: cyberbullying, and ⑦: other.

**Table 8:** Relationship between Perpetrators and Victims by Year.

Year	①	②	③	④	⑤	⑥	Total	*χ* ^2^	*p*
2018	309 (23.5%)	136 (10.3%)	5 (0.4%)	198 (15.1%)	169 (12.9%)	498 (37.9%)	1315 (100.0%)	964.46 ***	0.000
2019	500 (24.0%)	227 (10.9%)	5 (0.2%)	331 (15.9%)	373 (17.9%)	651 (31.2%)	2087 (100.0%)		
2020	429 (2.6%)	227 (4.6%)	15 (0.3%)	1239 (24.9%)	290 (5.6%)	2783 (56.0%)	4973 (100.0%)		
	1238 (14.8%)	590 (7.0%)	25 (0.3%)	1768 (21.1%)	822 (9.8%)	3932 (46.9%)	8375 (100.0%)		

*** *p* < 0.001. Where ①: close relationship, ②: social relationship, ③: family relationship ④: temporary relationship, ⑤: strangers, ⑥: unknown.

**Table 9:** Digital Sex Crimes by the Cognitive Path of Damage.

Year	Direct Recognition	Recognition by Others	Unknown	Total	*χ* ^2^	*p*
2018	561 (42.7%)	305 (23.2%)	449 (34.1%)	1315 (100.0%)	21.14 ***	0.000
2019	939 (45.0%)	434 (20.8%)	714 (34.2%)	2087 (100.0%)		
2020	1980 (39.8%)	1092 (22.0%)	1901 (38.2%)	4973 (100.0%)		
Total	3480 (41.6%)	1831 (21.9%)	3064 (36.6%)	8375 (100.0%)		

*** *p <* 0.001.

## Data Availability

Publicly available datasets were analyzed in this study. This data can be found here: (http://www.stop.or.kr) (accessed on 23 September 2021).
